# Gluon fusion into Higgs pairs at NLO QCD and the top mass scheme

**DOI:** 10.1140/epjc/s10052-019-6973-3

**Published:** 2019-05-30

**Authors:** J. Baglio, F. Campanario, S. Glaus, M. Mühlleitner, M. Spira, J. Streicher

**Affiliations:** 1 Institute for Theoretical Physics, Eberhard Karls Universität Tübingen, Auf der Morgenstelle 14, 72076 Tübingen, Germany; 2 Theory Division, IFIC, University of Valencia-CSIC, 46980 Paterna, Valencia Spain; 3 Institute for Theoretical Physics, Karlsruhe Institute of Technology, 76128 Karlsruhe, Germany; 4 Theory Group LTP, Paul Scherrer Institut, PSI, 5232 Villigen, Switzerland; 5 Institut für Theoretische Physik, Zürich University, 8057 Zurich, Switzerland

## Abstract

We present the calculation of the full next-to-leading order (NLO) QCD corrections to Higgs boson pair production via gluon fusion at the LHC, including the exact top-mass dependence in the two-loop virtual and one-loop real corrections. This is the first independent cross-check of the NLO QCD corrections presented in the literature before. Our calculation relies on numerical integrations of Feynman integrals, stabilised with integration-by-parts and a Richardson extrapolation to the narrow width approximation. We present results for the total cross section as well as for the invariant Higgs-pair-mass distribution at the LHC, including for the first time a study of the uncertainty due to the scheme and scale choice for the top mass in the loops.

## Introduction

Since the discovery of a Higgs boson at the LHC [1, 2], an enormous amount of data has been collected to analyse the properties of this newly discovered particle. Up to now the Standard Model (SM) Higgs boson hypothesis [3–6] is the most favoured one, even if there is still some room for new physics effects in the Higgs-coupling measurements. The experimental determination of the Higgs boson self-couplings, one of the most important measurements in the Higgs sector and a major goal of the high-luminosity upgrade of the LHC and of future high-energy colliders, is still yet to be performed and would give access to the scalar potential itself which is at the origin of the electroweak symmetry breaking mechanism. In order to obtain the Higgs self-couplings and in particular the triple Higgs coupling $\lambda _{H^3}$, the pair production of Higgs bosons needs to be considered [7, 8]. For the energies reachable at the LHC the measurement of the quartic Higgs self-coupling will be hopeless, since the triple Higgs-production cross section is too small [9–11].

The dominant production channel for Higgs pair production at hadron colliders is gluon fusion. It is mediated by heavy quark-loops at leading order (LO), in two different topologies: triangle diagrams containing the triple Higgs coupling, and box diagrams as an irreducible “background” for the extraction of $\lambda _{H^3}$ [12–15]. The NLO QCD corrections have been calculated in the heavy top-quark limit (HTL) some time ago [7], a framework in which the leading term of a heavy top mass expansion is obtained so that the NLO calculation reduces to one-loop corrections to effective *HHg* and *HHgg* couplings. The *K*–factor is found to be as sizeable as the corresponding *K*–factor for single Higgs production, $K\lesssim 2$. The next-to-next-to-leading order (NNLO) QCD corrections in the same HTL approximation have been computed in Refs. [16–18] for the total cross section and found to be of the order of $\simeq +20\%$, and in Ref. [19] for the differential distributions. Soft gluon resummation at next-to-next-to-leading logarithmic (NNLL) accuracy has been performed [20, 21], and the 3-loop matching coefficient has been derived in Refs. [18, 22].

There has been an enormous effort in the past few years to reach the full NLO QCD accuracy in Higgs pair production via gluon fusion including finite top mass $m_t$ effects. After the calculation of $m_t$-effects in the real radiation [23, 24] leading to a $-10\%$ reduction of the cross section, a $1/m_t$ expansion up to the order $\mathscr {O}(1/m_t^{12})$ lead to an estimate of the mass effects at the order of $\pm 10\%$ for the total cross section at NLO QCD [25, 26]. The first calculation of the full NLO QCD corrections has been performed in [27, 28] applying numerical methods based on sector decomposition and contour deformation to master integrals, and has shown that the $m_t$-effects in the virtual corrections are of the order of $\sim -5\%$ for the total cross section, but can amount to $\sim -25\%$ in the tail of the invariant Higgs-pair-mass ($m_{HH}$) distribution. This fixed-order calculation has been matched to parton shower programs later [29, 30] and combined with the NNLO QCD corrections in the HTL in Ref. [31]. Up to now no other independent calculation of the full NLO QCD corrections has been available, but several approximations have been able to partially reproduce the mass effects in the virtual corrections [32, 33]. Analytic results in the high-energy limit are also available in Ref. [34].

This letter presents the first independent calculation of the top-mass effects at NLO QCD for Higgs boson pair production via gluon fusion at the LHC since the original work of Refs. [27, 28]. Our method is based on the direct numerical integration of the Feynman integrals of the full diagrams, using integration-by-parts to improve the stability beyond the virtual thresholds, defined by intermediate gluon pairs and top-quark pairs, and a Richardson extrapolation [35] to obtain the final numbers in the narrow-width approximation of the virtual top quarks. We will present our results for the total cross section and for the invariant Higgs-pair-mass distribution at a center-of-mass (cm) energy of 14 TeV. We will perform for the first time an analysis of the uncertainties due to the scheme and scale chosen for the top mass.

The paper is organised as follows. In Sect. 2 we will describe the technical details of our calculation. In Sect. 3 we will present our numerical results. We present the renormalisation and factorisation scale uncertainties in Sect. 4 and derive the uncertainty due to the top mass in Sect. 5. In Sect. 6 we will close with our conclusions.

## Calculation

### Partonic leading order cross section

At LO, the gluon fusion process is mediated by heavy quark loops. There are triangle diagrams involving the triple Higgs coupling and box diagrams with two Yukawa couplings. As the Yukawa coupling is proportional to the mass of the quarks in the loop, we only consider the top-quark contribution. Following the conventions of Ref. [7], the cross section can be decomposed into form factors after applying two tensor projectors on the matrix elements, leading to the following LO expression for the partonic cross section $\hat{\sigma }(gg\rightarrow HH)$,

1 $$\begin{aligned} \hat{\sigma }_\mathrm{LO} = \frac{G_F^2 \alpha _s^2(\mu _R^2)}{256\left( 2\pi \right) ^3} \int _{\hat{t}_-}^{\hat{t}^+} d\hat{t}\, \left[ \left| C_\triangle ^{} F_\triangle ^{} + F_\square ^{} \right| ^2 + \left| G_\square ^{} \right| ^2\right] , \end{aligned}$$

where $G_F=1.1663787\times 10^{-5}\,\mathrm {GeV^{-2}}$ is the Fermi constant, $\alpha _s(\mu _R^2)$ is the strong coupling constant evaluated at the renormalisation scale $\mu _R$, and the Mandelstam variables $\hat{s}$ and $\hat{t}$ are given by

2 $$\begin{aligned} \hat{s}&= Q^2 = m_{HH}^2,\nonumber \\ \hat{t}&= -\frac{1}{2} \left[ Q^2 - 2 m_H^2 - Q^2\sqrt{1-\frac{4 m_H^2}{Q^2}}\, \cos \theta \right] , \end{aligned}$$

with the scattering angle $\theta$ in the partonic c.m. system and where $m_H$ is the Higgs boson mass. The integrations limits are given by

3 $$\begin{aligned} \hat{t}_\pm = -\frac{1}{2} \left[ Q^2 - 2 m_H^2 \mp Q^2\sqrt{1-\frac{4 m_H^2}{Q^2}}\, \right] . \end{aligned}$$

The coefficient $C_\triangle ^{}= \lambda _{H^3}v/(Q^2-m_H^2) = 3 m_H^2/(Q^2-m_H^2)$ contains $\lambda _{H^3}$ with $v\simeq 246$ GeV being the vacuum expectation value of the Higgs field, and the form factors reduce to $F_\triangle ^{} = - F_\square ^{} = 2/3$ and $G_\square ^{} = 0$ in the HTL approximation. The full $m_t$-dependence can be found in Refs. [13, 15].

### Hadronic cross section

**Fig. 1:**
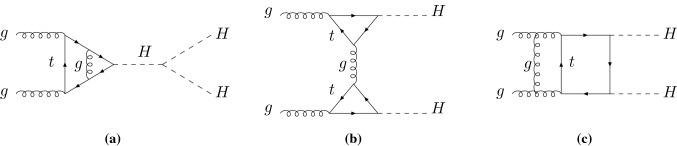
Typical two-loop diagrams contributing to Higgs-boson pair production via gluon fusion: **a** triangle diagram, **b** one-particle reducible diagram, **c** box diagram

The NLO QCD corrections include the two-loop virtual corrections to the triangle and box diagrams, the virtual one-particle-reducible double-triangle diagrams, and the one-loop $2\rightarrow 3$ real corrections. All these contributions are convolved with the parton distributions functions (PDFs) $f_{i}$ evaluated at the factorisation scale $\mu _F$, that are included in the parton luminosities $d\mathscr {L}^{ij}/d\tau$,

4 $$\begin{aligned} \frac{d\mathscr {L}^{i j}}{d\tau } = \int _\tau ^1 \frac{dx}{x} f_i\left( x,\mu _F\right) f_j\left( \frac{\tau }{x},\mu _F\right) , \end{aligned}$$

with $\tau =Q^2/s$, *s* being the hadronic cm energy. The hadronic cross section can be cast into the form

5 $$\begin{aligned} \sigma _\mathrm{NLO} = \sigma _\mathrm{LO} + \varDelta \sigma _\mathrm{virt} + \varDelta \sigma _{gg} + \varDelta \sigma _{qg} + \varDelta \sigma _{q\bar{q}}, \end{aligned}$$

with [7]

6 $$\begin{aligned} \sigma _\mathrm{LO}&= \int _{\tau _0}^1 d\tau \frac{d\mathscr {L}^{gg}}{d\tau } \hat{\sigma }_\mathrm{LO}\left( Q^2 = \tau s\right) ,\nonumber \\ \varDelta \sigma _\mathrm{virt}&= \frac{\alpha _s\left( \mu _R^2\right) }{\pi }\int _{\tau _0}^1 d\tau \frac{d\mathscr {L}^{gg}}{d\tau } \hat{\sigma }_\mathrm{LO}\left( Q^2 = \tau s\right) \, C_\mathrm{virt}\left( Q^2\right) ,\nonumber \\ \varDelta \sigma _{i j}&= \frac{\alpha _s\left( \mu _R^2\right) }{\pi }\int _{\tau _0}^1 d\tau \frac{d\mathscr {L}^{i j}}{d\tau } \int _{\frac{\tau _0}{\tau }}^1 \frac{dz}{z}\, \hat{\sigma }_\mathrm{LO}\left( Q^2 = z\tau s\right) C_{i j}(z), \end{aligned}$$

for $ij = gg$, $\displaystyle \sum _{q,\bar{q}} qg$, and $\displaystyle \sum _q q\bar{q}$, $z=Q^2/\tau s$, and $\tau _0 = 4 m_H^2/s$. We include five external massless quark flavours. The coefficients $C_{virt}$ of the virtual and $C_{ij}$ of the real corrections in the HTL have been obtained in Ref. [7] and are given by

7 $$\begin{aligned} C_{virt}&= \frac{11}{2} + \pi ^2 + C^\infty _{\triangle \triangle } + \frac{33-2N_F}{6} \log \frac{\mu _R^2}{Q^2}, \nonumber \\ C_{\triangle \triangle }&= \mathfrak {R}e~\frac{\int _{\hat{t}_-}^{\hat{t}_+} d\hat{t} \left\{ c_1 \Big [ (C_\triangle F_\triangle + F_\Box ) + \frac{p_T^2}{\hat{t}} G_\Box \Big ] + (\hat{t} \leftrightarrow \hat{u}) \right\} }{\int _{\hat{t}_-}^{\hat{t}_+} d\hat{t} \left\{ |C_\triangle F_\triangle + F_\Box |^2 + |G_\Box |^2 \right\} }, \nonumber \\ C^\infty _{\triangle \triangle }&= \left. C_{\triangle \triangle } \right| _{c_1 = 2/9}, \nonumber \\ C_{gg}&= -z P_{gg}(z) \log \frac{\mu _F^2}{\tau s} - \frac{11}{2} (1-z)^3 \nonumber \\&\quad + 6[1+z^4+(1-z)^4] \left( \frac{\log (1-z)}{1-z}\right) _+, \nonumber \\ C_{gq}&= -\frac{z}{2} P_{gq}(z) \log \frac{\mu _F^2}{\tau s (1-z)^2} + \frac{2}{3} z^2 - (1-z)^2, \nonumber \\ C_{q\bar{q}}&= \frac{32}{27} (1-z)^3, \end{aligned}$$

where $C^\infty _{\triangle \triangle }$ denotes the contribution of the one-particle reducible diagrams (see Fig. 1b) in the HTL with the transverse momentum $p_T^2 = (\hat{t}\hat{u} - m_H^4)/Q^2$ involving $\hat{u} = 2m_H^2 - Q^2 - \hat{t}$. $P_{gg}(z)$ and $P_{gq}(z)$ are the corresponding Altarelli-Parisi splitting functions [36], given by

8 $$\begin{aligned} P_{gg}(z)&= 6\left\{ \left( \frac{1}{1-z}\right) _+ +\frac{1}{z}-2+z(1-z) \right\} \nonumber \\&\quad + \frac{33-2N_F}{6}\delta (1-z),\nonumber \\ P_{gq}(z)&= \frac{4}{3} \frac{1+(1-z)^2}{z}, \end{aligned}$$

with $N_F=5$ in our calculation. For the LO cross section $\hat{\sigma }_\mathrm{LO}(Q^2)$ the full quark-mass dependence is taken into account at the integrand-level. These expressions can easily be converted into the differential cross section with respect to the invariant Higgs-pair mass,

9 $$\begin{aligned} \frac{d\sigma _\mathrm{LO}}{dQ^2}&= \left. \frac{d\mathscr {L}^{gg}}{d\tau } \frac{\hat{\sigma }_\mathrm{LO}\left( Q^2\right) }{s} \right| _{\tau = \frac{Q^2}{s}},\nonumber \\ \frac{d\varDelta \sigma _\mathrm{virt}}{dQ^2}&= \left. \frac{\alpha _s\left( \mu _R^2\right) }{\pi } \frac{d\mathscr {L}^{gg}}{d\tau } \frac{\hat{\sigma }_\mathrm{LO}\left( Q^2 \right) }{s}\, C_\mathrm{virt}\left( Q^2\right) \right| _{\tau = \frac{Q^2}{s}},\nonumber \\ \frac{d\varDelta \sigma _{i j}}{dQ^2}&= \left. \frac{\alpha _s\left( \mu _R^2\right) }{\pi }\int _{\frac{Q^2}{s}}^1 \frac{dz}{z^2} \frac{d\mathscr {L}^{i j}}{d\tau } \, \frac{\hat{\sigma }_\mathrm{LO}\left( Q^2\right) }{s} C_{i j}(z) \right| _{\tau = \frac{Q^2}{zs}}. \end{aligned}$$

### Virtual corrections

The two-loop virtual corrections split into three different types of contributions: triangle diagrams with a virtual Higgs propagator in the s-channel, box diagrams, and one-particle reducible diagrams. Typical examples of these three classes are shown in Fig. 1. According to Eq. (9) the two-loop triangle diagrams coincide with the production of a single Higgs boson with virtuality $Q^2$ so that they can be taken from this NLO calculation [37–41]. On the other hand the one-particle reducible diagrams involving two triangle loops of the Higgs coupling to one on-shell and one off-shell gluon can be constructed from the LO (one-loop) calculation of the Higgs decay $H\rightarrow Z\gamma$ [42, 43] where the couplings and colour factors have to be adjusted appropriately and the *Z* mass has to be replaced by the virtuality of the gluon in the t/u-channel. This results in the one-particle reducible contribution to the virtual corrections

10 $$\begin{aligned} c_1&= 2 \Big [ I_1(\tau ,\lambda _{\hat{t}}) -I_2(\tau ,\lambda _{\hat{t}}) \Big ]^2, \nonumber \\ I_1(\tau ,\lambda )&= \frac{\tau \lambda }{2(\tau -\lambda )} + \frac{\tau ^2\lambda ^2}{2(\tau -\lambda )^2} \left[ f(\tau ) - f(\lambda )\right] \nonumber \\&\quad + \frac{\tau ^2\lambda }{(\tau -\lambda )^2} \left[ g(\tau ) - g(\lambda ) \right] , \nonumber \\ I_2(\tau ,\lambda )&= - \frac{\tau \lambda }{2(\tau -\lambda )}\left[ f(\tau ) - f(\lambda ) \right] , \end{aligned}$$

with $\tau = 4m_t^2/m_H^2$, $\lambda _{\hat{t}} = 4 m_t^2/\hat{t}$ and the functions

11 $$\begin{aligned} f(\tau )&= \left\{ \begin{array}{ll} \displaystyle \arcsin ^2 \frac{1}{\sqrt{\tau }} &{} \tau \ge 1 \\ \displaystyle - \frac{1}{4} \left[ \log \frac{1+\sqrt{1-\tau }}{1-\sqrt{1-\tau }} - i\pi \right] ^2 &{} \tau< 1 \end{array} \right. \, , \nonumber \\ g(\tau )&= \left\{ \begin{array}{ll} \displaystyle \sqrt{\tau -1} \arcsin \frac{1}{\sqrt{\tau }} &{} \tau \ge 1\\ \displaystyle \frac{\sqrt{1-\tau }}{2} \left[ \log \frac{1+\sqrt{1-\tau }}{1-\sqrt{1-\tau }} - i\pi \right] &{} \tau < 1 \end{array} \right. \,. \end{aligned}$$

This expression has to be inserted in the $C_{\triangle \triangle }$ coefficient of Eq. (7) and agrees with the previous analytical result of Ref. [44].

The cumbersome part of this calculation is the computation of the two-loop box diagrams. We have performed a Feynman parametrisation of the full virtual diagrams individually without any tensor reduction to master integrals. The ultraviolet singularities are extracted by appropriate end-point subtractions. Special care, however, is required for the infrared divergent diagrams of the type of e.g. Fig. 1c that correspond to gluon-rescattering involving a threshold starting at $Q^2=0$, i.e. in the whole kinematical range of the process. The Feynman parametrisation can be adjusted such that the (singular) denominator is expressed as a second-order polynomial in terms of one of the Feynman parameters (here $x_6$, while $x_1,\ldots ,x_5$ correspond to the other parameters),

12 $$\begin{aligned} I&= \int d^5 \mathbf {x} dx_6 \frac{x_6^{1+\epsilon } H(\mathbf {x},x_6)}{N(\mathbf {x},x_6)^{3+2\epsilon }} \qquad [\mathbf {x} = (x_1,\ldots ,x_5)], \nonumber \\ N(\mathbf {x},x_6)&= a x_6^2 + b x_6 + c, \end{aligned}$$

where $H(\mathbf {x},x_6)$ denotes the numerator related to the full spinorial structure of the diagram and

13 $$\begin{aligned} a,c = \mathcal{O} \left( \frac{1}{m_t^2}\right) , \qquad b = 1 + \mathcal{O} \left( \frac{1}{m_t^2} \right) , \end{aligned}$$

where the coefficient *c* can be expressed as a pure product of a kinematical ratio of $\mathcal{O} (1/m_t^2)$ and other Feynman parameters. Based on the fact that the relative infrared singularities are universal we constructed a subtraction term by keeping only *b* and *c* in the denominator $N_0(\mathbf {x},x_6) = b x_6 + c$ thus rewriting the integral of Eq. (12) as

14 $$\begin{aligned} I&= I_1 + I_2, \nonumber \\ I_1&= \int d^5 \mathbf {x} dx_6 \left\{ \frac{x_6^{1+\epsilon } H(\mathbf {x},x_6)}{N(\mathbf {x},x_6)^{3+2\epsilon }} - \frac{x_6^{1+\epsilon } H(\mathbf {x},0)}{N_0(\mathbf {x},x_6)^{3+2\epsilon }} \right\} ,\nonumber \\ I_2&= \int d^5 \mathbf {x} dx_6 \frac{x_6^{1+\epsilon } H(\mathbf {x},0)}{N_0(\mathbf {x},x_6)^{3+2\epsilon }}. \end{aligned}$$

The integral $I_1$ can now be expanded in $\epsilon$ leading to numerically finite integrals for each expansion coefficient, while the integral $I_2$ can be integrated over $x_6$ yielding hypergeometric functions. The transformation properties of the latter (related to an inversion of the kinematical argument) allow for a clean separation of the infrared singularities. The remaining singularities in the coefficient *c* can be treated by end-point subtractions. The cumbersome treatment of these diagrams can be related to the appearance of two different scales, $m_t$ and *Q*, that control the high-scale and low-scale parts of the whole calculation in the sense of an effective theory, the HTL, where the high scale is given by the top mass and the low scale by *Q*. This method of constructing the infrared subtraction term has been developed within the old NLO calculation of single Higgs production [37, 38] for internal numerical checks. Since the integration over $\hat{t}$ is not finite for the individual two-loop diagrams we have introduced a cut at the integration bounds as well as a suitable substitution to stabilise the integration close to the bounds. By varying the cut around its central value we have checked that the total sum of all two-loop box diagrams is finite and independent of the particular value of this cut.

For the analytical continuation of our virtual amplitudes above the thresholds we introduced a small imaginary part of the virtual top mass, $m_t^2 \rightarrow m_t^2 (1-i\bar{\epsilon })$ in our numerical integration. However, to stabilise the numerical integration above the thresholds we had to perform integration by parts in one of the involved Feynman parameters in order to reduce the power of the corresponding denominator for each diagram individually. In this way we achieved a reliable accuracy of our numerical integrations for $\bar{\epsilon }$ values down to about 0.1. In order to obtain the result in the narrow-width approximation ($\bar{\epsilon }\rightarrow 0$) we performed a Richardson extrapolation [35] applied to the results for different values of $\bar{\epsilon }$.^1^ In particular, we use $\bar{\epsilon }$ in the range given by $\bar{\epsilon }_n= 0.05\times 2^n$, with $n=0,\ldots ,9$. In the dominant region, we use the set $n=1,\dots ,9$, with the exception of the bins in the range $Q\in [300-475]$ GeV where the complete set of values is used. Starting at $Q=700$ GeV, we restrict ourselves to values in the range $n=1,\ldots ,5$. This allows us to obtain a series of extrapolated results up to the ninth order in the dominant region and up to the fifth order in the tails. We define a theoretical error estimate due to the Richardson extrapolation as the difference of the fifth and the fourth order extrapolated results. Moreover, this error is multiplied by a factor of two close to the top threshold, in order to be conservative. The obtained estimated Richardson extrapolated error falls below the percent level of accuracy and is added in quadrature to the statistical integrated error.

The top mass has been renormalised in the on-shell scheme and the strong coupling constant in the $\overline{\mathrm{MS}}$ scheme with 5 active flavours, i.e. with a decoupled top quark. We have achieved a finite result for the virtual corrections by subtracting the virtual correction in the HTL so that our numerical integration yields the NLO mass effects only. The virtual corrections in the HTL have then been added back by the results of HPAIR.^2^ The calculation of each two-loop box diagram has been performed independently at least twice with different Feynman parametrisations.

### Real corrections

We are left with the calculation of the coefficients $C_{ij}$, or more specifically the calculation of the finite $m_t$-effects in the real corrections, $\varDelta \sigma _{ij}^\mathrm{mass} = \varDelta \sigma _{ij} - \varDelta \sigma _{ij}^\mathrm{HTL}$, where $\varDelta \sigma _{ij}^\mathrm{HTL}$ involves the full mass-dependent LO contribution as exemplified in Eqs. (6, 9). The HTL expressions for the coefficients $C_{ij}$ are given in Eq. (7) [7] and can be calculated using the program HPAIR.

To obtain the mass effects, we use the fact that the infrared divergences in the real corrections are universal and are the same in the full calculation and in the HTL approximation. At any given phase-space point we can subtract the HTL result from the full calculation, obtaining an infrared-finite result which is exactly the remainder due to the mass effects in the full real corrections,

15 $$\begin{aligned} d\varDelta \hat{\sigma }_{ij}^\mathrm{mass} = d\varDelta \hat{\sigma }_{ij} - d\hat{\sigma }_\mathrm{LO}(\tilde{p}_i) \frac{d\varDelta \hat{\sigma }_{ij}^\mathrm{HTL}(p_i)}{d\hat{\sigma }_\mathrm{LO}^\mathrm{HTL}(\tilde{p}_i)}. \end{aligned}$$

In order to calculate the LO matrix elements we need to map the full $2\rightarrow 3$ phase-space onto an on-shell LO $2\rightarrow 2$ sub-space, leading to a transformation of the 4-momenta $p_i \rightarrow \tilde{p}_i$. For this purpose, we use the mapping of Ref. [45] for the case of initial-state emitter and initial-state spectator to build our local subtraction term. The HTL matrix elements are calculated analytically. In order to achieve a good numerical stability we have implemented a technical collinear cut in the scattering angle integration of the extra parton in the final state, $|\cos \theta | < 1 - \delta$, with $\delta = 10^{-4}$. It has been checked that this value does not affect the physical results by varying $\delta$ around our nominal choice.

The full one-loop matrix elements contain triangle, box, and pentagon diagrams. They are generated using Feyn-Arts [46] and FormCalc [47], while the scalar one-loop integrals are numerically calculated using the library COLLIER 1.2 [48] interfaced to the output of FormCalc with an in-house library. We have performed two independent calculations with different parametrisations of the $2\rightarrow 3$ phase-space and different versions of FeynArts and FormCalc. We have also cross-checked the mass effects of the real corrections against Refs. [23, 24, 27, 28] and we have obtained mutual agreement.

**Fig. 2:**
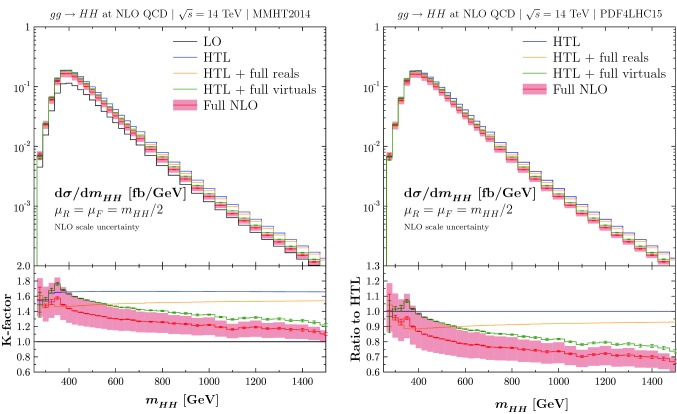
Invariant Higgs-pair-mass distributions for Higgs boson pair production via gluon fusion at the 14 TeV LHC as a function of $Q=m_{HH}$. LO results (in black), HTL results (in blue), HTL results including the full real corrections (in yellow), HTL results including the full virtual corrections (in green, including the numerical error), and the full NLO QCD results (in red, including the numerical error). Left: Results with the MMHT2014 PDF set, the insert below displays the *K*-factors for the different results. Right: Results with the PDF4LHC15 PDF set, the insert below displays the ratio to the NLO HTL result for the different calculations. The red band indicates the renormalisation and factorisation scale uncertainties for results including the full NLO QCD corrections

## Numerical results

We present our numerical results at the LHC for a c.m. energy of $\sqrt{s}=14$ TeV. We use $m_H = 125$ GeV and $m_t=172.5$ GeV. We have performed the calculation using two NLO PDF sets, MMHT2014 [49] and PDF4LHC15 [50] as implemented in the LHAPDF-6 library [51]. Our central scale choice is $\mu _R=\mu _F=\mu _0 = m_{HH}/2$, and $\alpha _s(M_Z^2)$ is set according to the PDF set chosen, with a NLO running in the five-flavour scheme. We recall that *Q* and $m_{HH}$ refer to the same physical quantity. We have performed the whole calculation twice independently using also different parametrisations of the virtual Feynman integrals and of the real phase-space. Both independent calculations agree with each other within the numerical errors. Note that our calculation has been performed in the narrow-width approximation for the top quark. The finite-width effect amounts to $\sim -2\%$ at LO for the total cross section, corresponding to a maximum deviation of $\sim -4\%$ at the $t\bar{t}$ threshold in the invariant Higgs-pair-mass distribution [24].

We have calculated a grid of *Q*-values from $Q=250~\mathrm {GeV}$ to $Q=1500~\mathrm {GeV}$, so that we obtain the invariant Higgs-pair-mass distribution depicted in Fig. 2. We compare our full results, displayed in red, to three different approximations: The HTL results according to Ref. [7] are displayed in blue; the HTL plus the mass effects in the real corrections only are shown in yellow; the HTL plus the mass effects in the virtual corrections only are presented in green. The error bars due to the total numerical errors are also given for the green and red histograms. They are determined from the individual integration errors and the errors due to the Richardson extrapolation – added in quadrature. The numerical errors are negligible for the other predictions (HTL and HTL plus the mass effects in the real corrections only). The red band indicates the renormalisation and factorisation scale uncertainties of our prediction for the full NLO QCD predictions, see next section for more details. In the case of the MMHT2014 PDF set we also compare to the LO results. We reproduce the results of Refs. [23, 24] for the mass effects in the real corrections, which are mildly varying from $m_{HH} = 400$ GeV to $m_{HH} = 1500$ GeV, and are of the order of $-10\%$. The global *K*-factor displayed on the left-hand-side of Fig. 2 is decreasing in the whole range and gets close to 1 for large $m_{HH}$ values. The mass effects in the virtual corrections reach $\simeq -20\%$ at large $m_{HH}$ values, in agreement with the findings of Refs. [27, 28]. The mass effects are negative in accordance with the expected restoration of partial-wave unitarity in the high-energy limit.

From the differential distribution we calculate the total cross section using a numerical integration over *Q* with the trapezoidal method supplemented by a Richardson extrapolation [35] for $Q>300$ GeV, while for $Q<300$ GeV we have used the extension of Boole’s rule to six nodes [52]. We obtain the following results for the NLO cross sections (the numbers in parenthesis indicate the numerical errors, i.e. the quadratic sum of the statistical and the Richardson extrapolation error),

16 $$\begin{aligned} \sigma _{gg\rightarrow HH}^\mathrm{PDF4LHC} = 32.78(7)\,\mathrm {fb}, \quad \sigma _{gg\rightarrow HH}^\mathrm{MMHT} = 33.33(7)\,\mathrm {fb}. \end{aligned}$$

These have to be compared to the HTL results,

17 $$\begin{aligned} \sigma _{gg\rightarrow HH}^\mathrm{HTL, PDF4LHC} = 38.66\,\mathrm {fb}, \quad \sigma _{gg\rightarrow HH}^\mathrm{HTL, MMHT} = 39.34\,\mathrm {fb}. \end{aligned}$$

Comparing Eqs. (16) and (17), we derive $\simeq -15\%$ top-mass effects at NLO on the total cross section. We are in mutual agreement with the results of Refs. [27, 28] within the numerical uncertainties and taking into account the small difference in the choice of the top mass value.

## Factorisation and renormalisation scale dependence

For the estimate of the residual theoretical uncertainties of our NLO results we derive the uncertainties due to the choices of the factorisation and renormalisation scales by varying both by a factor of two up and down around the central scale choice $\mu _R=\mu _F=m_{HH}/2$, but avoid a splitting of both scales by more than a factor of two. This leads to seven points in the $\mu _R-\mu _F$-plane, since both scale dependences are monotonic. The scale uncertainty is then determined by the maximal and minimal cross section values around the central values. The factorisation scale enters the luminosity factors and the HTL parts of the coefficients $C_{ij}$ in Eq. (9) so that the numerical two-loop results of the NLO mass effects are not affected. The same is true for the renormalisation scale dependence, since the strong coupling constant enters only as a universal factor in each perturbative order and the explicit renormalisation scale appears only in the HTL-part of the virtual corrections. In total we find the following scale dependences for the differential cross section for four distinct values of *Q*,

18 $$\begin{aligned} \frac{d\sigma (gg\rightarrow HH)}{dQ}\Big |_{Q=300~\mathrm{GeV}}&= 0.0298(7)^{+15.3\%}_{-13.0\%}\, \mathrm {fb/GeV},\nonumber \\ \frac{d\sigma (gg\rightarrow HH)}{dQ}\Big |_{Q=400~\mathrm{GeV}}&= 0.1609(4)^{+14.4\%}_{-12.8\%}\, \mathrm {fb/GeV},\nonumber \\ \frac{d\sigma (gg\rightarrow HH)}{dQ}\Big |_{Q=600~\mathrm{GeV}}&= 0.03204(9)^{+10.9\%}_{-11.5\%}\, \mathrm {fb/GeV},\nonumber \\ \frac{d\sigma (gg\rightarrow HH)}{dQ}\Big |_{Q=1200~\mathrm{GeV}}&= 0.000435(4)^{+7.1\%}_{-10.6\%}\, \mathrm {fb/GeV}, \end{aligned}$$

for PDF4LHC parton densities, while the total cross section develops the uncertainties

19 $$\begin{aligned} \sigma (gg\rightarrow HH) = 32.78(7)^{+13.5\%}_{-12.5\%}\,\mathrm {fb}. \end{aligned}$$

The last result is in mutual agreement with Refs. [27, 28] within the numerical uncertainties and taking into account the small difference in the choice of the top mass value.

## Uncertainty due to the top mass scheme

For the uncertainty related to the scheme and scale choice of the top mass we have calculated the total NLO results for the differential gluon-fusion cross section for the $\overline{\mathrm{MS}}$ top mass at different scale choices and have compared to our default prediction using the top quark pole mass both in the loop propagators and in the Yukawa coupling. We have used an N$^3$LO evolution and conversion of the pole into the $\overline{\mathrm{MS}}$ mass at the input scale given by the $\overline{\mathrm{MS}}$ top mass itself. This leads, for our choice of $m_t=172.5$ GeV for the top pole mass to an $\overline{\mathrm{MS}}$ mass of $\overline{m}_t(\overline{m}_t) = 163.02$ GeV. The renormalisation of the top mass has been adjusted accordingly in our calculation, and we have switched to an $\overline{\mathrm{MS}}$ mass both in the loop propagators and in the Yukawa coupling. We present the top-quark scheme uncertainties at four selected values of *Q* in the invariant Higgs-pair mass differential cross section. We take the maximum and minimum differential cross sections when the scale of the $\overline{\mathrm{MS}}$ top quark mass is varied in the range *Q* / 4 and *Q*, compared to our default pole mass predictions, and we obtain the following variations,

20 $$\begin{aligned} \frac{d\sigma (gg\rightarrow HH)}{dQ}\Big |_{Q=300~\mathrm{GeV}}&= 0.0298(7)^{+6\%}_{-34\%}\, \mathrm {fb/GeV},\nonumber \\ \frac{d\sigma (gg\rightarrow HH)}{dQ}\Big |_{Q=400~\mathrm{GeV}}&= 0.1609(4)^{+0\%}_{-13\%}\, \mathrm {fb/GeV},\nonumber \\ \frac{d\sigma (gg\rightarrow HH)}{dQ}\Big |_{Q=600~\mathrm{GeV}}&= 0.03204(9)^{+0\%}_{-30\%}\, \mathrm {fb/GeV},\nonumber \\ \frac{d\sigma (gg\rightarrow HH)}{dQ}\Big |_{Q=1200~\mathrm{GeV}}&= 0.000435(4)^{+0\%}_{-35\%}\, \mathrm {fb/GeV}, \end{aligned}$$

using PDF4LHC parton densities. The top-quark scheme uncertainty is significant over the whole range of $m_{HH}$. Note that a similar result has been observed in single Higgs production for large Higgs masses which correspond to our triangle diagram involving the triple Higgs coupling. Furthermore, this scheme uncertainty is reduced by roughly a factor of two from LO to NLO. The prediction involving the top pole mass, that we take as our central prediction, is the maximal prediction for high $m_{HH}$ values. The uncertainties induced by the top-mass scheme and scale choice on the total cross section at NLO will be given in a forthcoming publication [53].

## Conclusions

We have presented the calculation of the full NLO QCD corrections to Higgs-boson pair production via gluon fusion for the top-loop contributions. This has been performed by numerical integrations of the involved virtual two-loop corrections to the four-point functions, while the results of the single-Higgs case have been translated to the three-point contributions that involve the trilinear Higgs self-coupling. The one-particle reducible contributions that appear for the first time at NLO have been inferred from the explicit analytical one-loop results for $H\rightarrow Z\gamma$, where the *Z*-boson mass plays the role of the virtuality of the gluon in the dressed $Hgg^*$ vertex. In order to isolate the ultraviolet, infrared and collinear divergences, we have performed appropriate end-point subtractions at the integrand level and described the explicit construction of infrared subtraction terms that allow for a clean separation of the infrared singularities from the regular rest. The real corrections have been obtained by generating the full matrix elements with automatic tools. We have constructed the infrared and collinear subtraction term as the heavy-top limit of the real matrix elements involving the fully massive LO sub-matrix element. Adding back the full results in the heavy-top limit completed the full real corrections. The final results we have obtained agree with previous calculations for the individual finite parts of the real and virtual corrections. We find finite NLO mass effects that are up to $-30\%$ for large invariant Higgs-pair masses, while the total NLO top-mass effects modify the total cross section by about $-15\%$.

We have studied the theoretical uncertainties related to variations of the renormalisation and factorisation scales and have found agreement with the previously known results finding uncertainties at the level of $10-15\%$. A novel outcome of our calculation is the additional uncertainty induced by the scheme and scale dependence of the top mass that can be significant, amounting to $+6\%/-34\%$ at $m_{HH}=300$ GeV and $+0\%/-35\%$ at $m_{HH} = 1200$ GeV. The induced uncertainty on the total cross section will be given in a forthcoming publication [53].

In the future we plan to extend our calculation to beyond-the-SM models as e.g. the 2HDM or MSSM.

## Data Availability

This manuscript has no associated data or the data will not be deposited. [Authors’ comment: The research in the manuscript is of theoretical nature and therefore does not use any experimental data which needs to be deposited. All the numerical results are displayed in the figures.]

## Footnotes

1 It should be noted that a Richardson extrapolation of the integrand *before* integration provides an alternative method to stabilise the numerical integration.

2 The program can be downloaded at http://tiger.web.psi.ch/hpair/.
